# Pharmacointeraction Network Models Predict Unknown Drug-Drug Interactions

**DOI:** 10.1371/journal.pone.0061468

**Published:** 2013-04-19

**Authors:** Aurel Cami, Shannon Manzi, Alana Arnold, Ben Y. Reis

**Affiliations:** 1 Division of Emergency Medicine, Boston Children’s Hospital, Boston, Massachusetts, United States of America; 2 Department of Pharmacy, Boston Children’s Hospital, Boston, Massachusetts, United States of America; 3 Department of Pediatrics, Harvard Medical School, Boston, Massachusetts, United States of America; Universidad de Malaga, Spain

## Abstract

Drug-drug interactions (DDIs) can lead to serious and potentially lethal adverse events. In recent years, several drugs have been withdrawn from the market due to interaction-related adverse events (AEs). Current methods for detecting DDIs rely on the accumulation of sufficient clinical evidence in the post-market stage – a lengthy process that often takes years, during which time numerous patients may suffer from the adverse effects of the DDI. Detection methods are further hindered by the extremely large combinatoric space of possible drug-drug-AE combinations. There is therefore a practical need for predictive tools that can identify potential DDIs years in advance, enabling drug safety professionals to better prioritize their limited investigative resources and take appropriate regulatory action. To meet this need, we describe Predictive Pharmacointeraction Networks (PPINs) – a novel approach that predicts unknown DDIs by exploiting the network structure of all known DDIs, together with other intrinsic and taxonomic properties of drugs and AEs. We constructed an 856-drug DDI network from a 2009 snapshot of a widely-used drug safety database, and used it to develop PPIN models for predicting future DDIs. We compared the DDIs predicted based solely on these 2009 data, with newly reported DDIs that appeared in a 2012 snapshot of the same database. Using a standard multivariate approach to combine predictors, the PPIN model achieved an AUROC (area under the receiver operating characteristic curve) of 0.81 with a sensitivity of 48% given a specificity of 90%. An analysis of DDIs by severity level revealed that the model was most effective for predicting “contraindicated” DDIs (AUROC = 0.92) and less effective for “minor” DDIs (AUROC = 0.63). These results indicate that network based methods can be useful for predicting unknown drug-drug interactions.

## Introduction

Adverse drug-drug interactions (DDIs) are a serious health threat that can result in significant morbidity and mortality. In recent years, several drugs have been withdrawn from the market because of serious interaction-related adverse events (AEs). For example, the antihistamine drug astemizole (Hismanal) and the gastrointestinal-disorders drug cisapride (Propulsid) were withdrawn from the market in 1999 and 2000, respectively, after it was determined that each could cause fatal arrhythmias when given in combination with certain other drugs [1], [2]. Similarly, the hypertension drug mibefradil (Posicor) was withdrawn from the market in 1998 because of interactions with a number of other drugs [3].

Drug-drug interactions may be categorized by various criteria, two important ones being the severity level and the underlying DDI mechanism. In terms of severity, DDIs are often categorized into minor, moderate and severe (or, major) [4]. Minor DDIs are considered of slight clinical significance and typically only call for routine patient monitoring, moderate DDIs have a higher clinical significance and may require dosage changes and closer monitoring, and major DDIs can lead to serious adverse effects and should typically be avoided. In terms of mechanism, DDIs can be broadly categorized as either pharmacokinetic or pharmacodynamic [4]–[7]. Pharmacokinetic DDIs occur when one drug interferes with the absorption, distribution, metabolism, or elimination of another drug, leading to changes in the plasma concentration of the affected drug. One of the largest groups of pharmacokinetic interactions are those caused by the inhibition or induction of cytochrome P450 (CYP) isozymes, which are involved in the metabolism of many drugs [4], [5]. Pharmacodynamic interactions occur when one drug interferes with a second drug at a target site, leading to additive or subtractive effects for the involved drugs [4], [7]. Although important, pharmacodynamic interactions make up a smaller class than pharmacokinetic interactions.

Ideally, the interactions of a new drug with existing drugs could be predicted in the early stages of discovery and development. Traditionally, early stage predictions have focused on pharmacokinetic DDIs and include a variety of *in silico* [8]–[11] and *in vitro* methods [5], [12]–[15]. Presently, for many types of pharmacokinetic interactions, early stage prediction can be highly sensitive and specific. On the other hand, for pharmacodynamic DDIs, although early-stage prediction is also routinely conducted, it is comparatively less effective [16], [17]. In the later pre-market stages, *in vivo* experiments [18] and clinical trials are employed to check the most important interactions predicted in the early stages. Notwithstanding this wide range of activities, many important DDIs can go undetected in the pre-market phase, as evidenced by interaction-related post-market warnings and withdrawals.

For the many DDIs that go undetected in the pre-market phase, early detection during the post-market phase could lead to the prevention of many potential AEs, either through the addition of an interaction warning on the drug label, or in extreme cases through drug withdrawal. A number of statistical methods exist for detecting whether the combination of two drugs is associated with an increased risk of certain AEs. These methods analyze post-market data, such as spontaneous reports, insurance claim databases or electronic medical records [19]–[22]. In order to identify potential safety issues, these detection methods rely on waiting for sufficient post-market evidence to accumulate – a process that can take years, during which time numerous people may be affected by the adverse interaction. Detection methods are further hindered by the vastness of the space of possible drug-drug-AE combinations. Therefore, there is a practical need for predictive tools that can identify potential DDIs years in advance.

To meet this need, we propose Predictive Pharmacointeraction Networks (PPINs). PPINs exploit the network structure formed by the set of known DDIs, as well as various intrinsic and taxonomic properties of drugs, in order to predict unknown DDIs. PPINs work by constructing a network of known DDIs, where “nodes” represent drugs and “edges” represent the known interactions between drugs. A predictive model is developed to predict the unknown edges, or DDIs. Such a “link-prediction” approach has been used in other applications, such as social, metabolic or food networks [23], [24]. Network analysis is well suited for the medical and pharmacological domain, where complex relationships exist among various entities, such as drugs, targets, and diseases [25]–[28]. A number of recent pharmacological studies have proposed interesting applications of network analysis, including characterizations of the network structure formed by known drug-drug interactions [29], [30]. Our prior work has focused on developing network-based models to predict unknown drug-adverse event associations [31]. To our knowledge, the current study is the first to use network-based models for predicting unknown DDIs. While detection of unknown AEs is a difficult challenge, detection of unknown DDIs is far more challenging as it involves relying on sufficient evidence accumulating within a much smaller sub-population that is simultaneously exposed to both drugs and presenting with the relevant AE. Since DDI’s can be more difficult to detect than AE’s, it is even more critical to develop effective methods for predicting DDIs years in advance. Prediction of DDIs is a challenging and unique problem that requires a tailored set of methods and predictors. Whereas in predictive bipartite drug-AE networks, each drug node can only be connected to an AE node, in the DDI network each drug node may potentially be connected with every other node in the network.

In this study, we evaluate the performance of a predictive network model using a simulated-prospective validation based on two chronologically separated snapshots of a widely used drug safety database. This validation method preserves the historical order in which the data became available, thereby enabling a realistic assessment of the model’s predictive power. The computational approach proposed here is intended as a complementary hypothesis-generation tool to help drug safety experts identify potential drug-drug interactions. The predicted DDIs can be used to guide follow-up investigation by drug safety experts.

## Results

**Figure 1:**
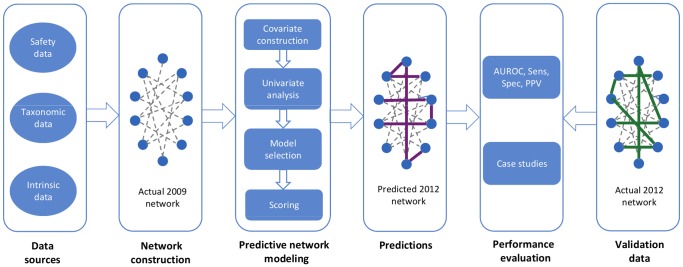
Overview of the PPIN approach for predicting drug-drug interactions (DDIs). Beginning on the left, data were integrated from multiple sources, including safety data (two snapshots of known DDIs from 2009 and 2012), taxonomic data (ATC taxonomy of drugs), and data related to the intrinsic properties of drugs (chemical substructures). Next, a network representation of the DDIs contained in the 2009 database snapshot was constructed from which a collection of network, taxonomic and intrinsic covariates were derived. These covariates were used to develop a predictive model based solely on the 2009 data that predicted new, unknown drug-drug interactions. These predictions were evaluated against the newly reported DDIs that appeared in the 2012 data.

We begin with an overview of the PPIN approach (Fig. 1), followed by a more detailed methodological account. To construct the PPIN, we integrated data from multiple sources, including DDI data, drug taxonomic data and intrinsic drug properties. Based on a 2009 snapshot of a widely used drug safety database, we constructed a network representation of all known DDIs, where each node represents a drug, and each edge connecting two nodes represents a known interaction between those two drugs. Next, we used these data to construct a set of covariates and to develop predictive logistic regression (LR) and generalized linear mixed (GLM) models. These models predicted the probabilities for all the non-edges of the 2009 network and those with the highest probabilities formed the model’s top predictions for unknown DDIs. We performed a simulated prospective evaluation of the model’s predictive performance by comparing these predictions with the set of newly reported DDIs that appeared in a 2012 version of the same drug safety database, and were not present in the 2009 snapshot.

All the above steps were also carried out for three sub-networks of the DDI network, namely the sub-networks induced by the “minor”, “major” and “contraindicated” DDIs. (Following standard network theory terminology, the subgraph induced by a set of edges consists of those edges together with any vertices that are their endpoints.).

### Data Description

**Figure 2:**
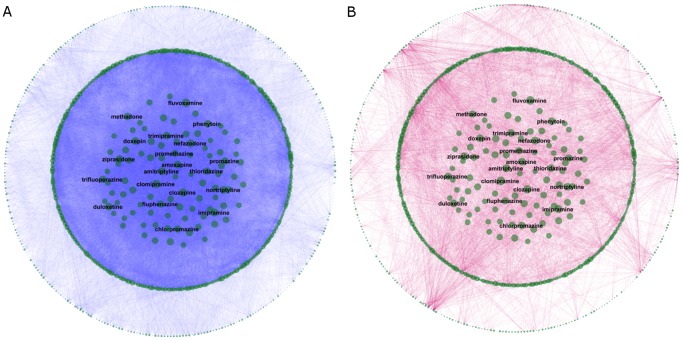
A visualization of the DDI network. (A) The DDIs present in the 2009 training dataset are shown in blue. (B) The DDIs newly reported in the 2012 validation dataset are shown in red. In both parts, the size of each node is proportional to the node degree in the 2009 network. Some of the most highly-connected drugs are labeled for illustrative purposes.

Fig. 2 provides a visualization of the DDI network (produced using the Cytoscape visualization tool, www.cytoscape.org). The data for constructing this network were obtained from the following sources: DDIs were extracted from two chronologically separated snapshots of Multum Vantage Rx, a widely used drug safety database. This database contains a variety of clinical information about drug products and diseases, including drug-drug interactions, drug-disease interactions, allergies, dosing information, and so on (http://www.multum.com/VantageRxDB.htm). In this study, we only used the Vantage Rx information on drug-drug interactions. The two snapshots used in the study contained all reported DDIs of all FDA-approved drugs as of October 2009 and March 2012, respectively. The taxonomic and intrinsic drug properties were extracted from World Health Organization Anatomical Therapeutic Chemical Classification System (ATC) (www.whocc.no/atc) and DrugBank (http://www.drugbank.ca/), respectively. Generic names were used to uniquely represent drugs and to perform data integration. A list of synonyms from NCGC Pharmaceutical Collection (NPC) [32] was used to identify the different forms of generic names that referred to a common “active pharmaceutical ingredient” (API).

After integrating the DDI data from the 2009 snapshot with the DrugBank and ATC drug data, we identified 856 unique drugs (APIs) for which valid data was available in all three databases. The 2009 data contained 55,560 DDIs formed by these drugs. The 2009 DDI network, thus, consisted of 856 nodes (drugs), 55,560 edges (DDIs) and 310,380 non-edges (pairs that were not known to form DDIs in 2009). The known DDIs in the training set made up 15.2% of all combinatorially possible DDIs. The 2012 DDI data snapshot reported 4,401 new DDIs among these 856 drugs (proportion of new DDIs in the validation set: 1.4% of all combinatorially possible DDIs that were not reported as DDIs in the 2009 dataset). Fig. 2A shows the DDIs contained in the 2009 safety database snapshot, while Fig. 2B shows the DDIs newly reported in the 2012 snapshot. In both parts of Fig. 2 node size is proportional to the node degree in the 2009 DDI network. Some of the drugs with high degree in 2009 are labeled for illustration purposes. As seen in Fig. 2B, many of the DDIs newly reported during 2009–2012 involved drugs that were already highly connected in 2009 (although a few drugs with small degree in 2009 had a notably large number of newly reported DDIs).

The 2009 sub-network induced by the set of “minor” DDIs (i.e. the minor-DDI sub-network) consisted of 696 drugs and 4,221 DDIs. In 2012, there were 271 newly reported “minor” DDIs among those drugs. The 2009 sub-network induced by the set of “major” DDIs (i.e. the major-DDI sub-network) consisted of 718 drugs and 7,263 DDIs. In 2012, there were 794 newly reported “major” DDIs among those drugs. The 2009 sub-network induced by the set of “contraindicated” DDIs consisted of 491 drugs and 2,323 DDIs. In 2012, there were 122 newly reported “contraindicated” DDIs among those drugs.

Using the 2009 DDI network we derived a number of network, taxonomic and intrinsic covariates (Table S1) corresponding to the drug pairs. The network covariates encode purely structural information contained in the DDI network, the taxonomic covariates encode information related to the ATC categories of drugs, while the intrinsic covariates encode information related to the molecular substructures of drugs. Three of the network covariates - degree_prod, betw_prod, and cccnw_max - aimed to capture, respectively, the popularity, centrality, and network clustering, which are widely used statistics in network analysis [33]. The remaining covariates aimed to capture the similarity (or dissimilarity) between the two drugs in terms of their network properties (jackard, jackard_max2_mean), taxonomic properties (atc_min, atc_min_prod), or chemical properties (str_jackard, str_max_prod). The similarity-based covariates are either 0^th^ or 1^st^ order: the computation of the 0^th^ order covariates relies only on the properties of the two drugs forming the pair (jackard, atc_min, str_jackard), whereas the computation of the 1^st^ order covariates relies on the properties of the two drugs forming the pair as well as on the properties of their network neighbors.

The above covariates were also computed for the 2009 minor-DDI, major-DDI, and contraindicated-DDI sub-networks.

### Predictive Performance

**Figure 3:**
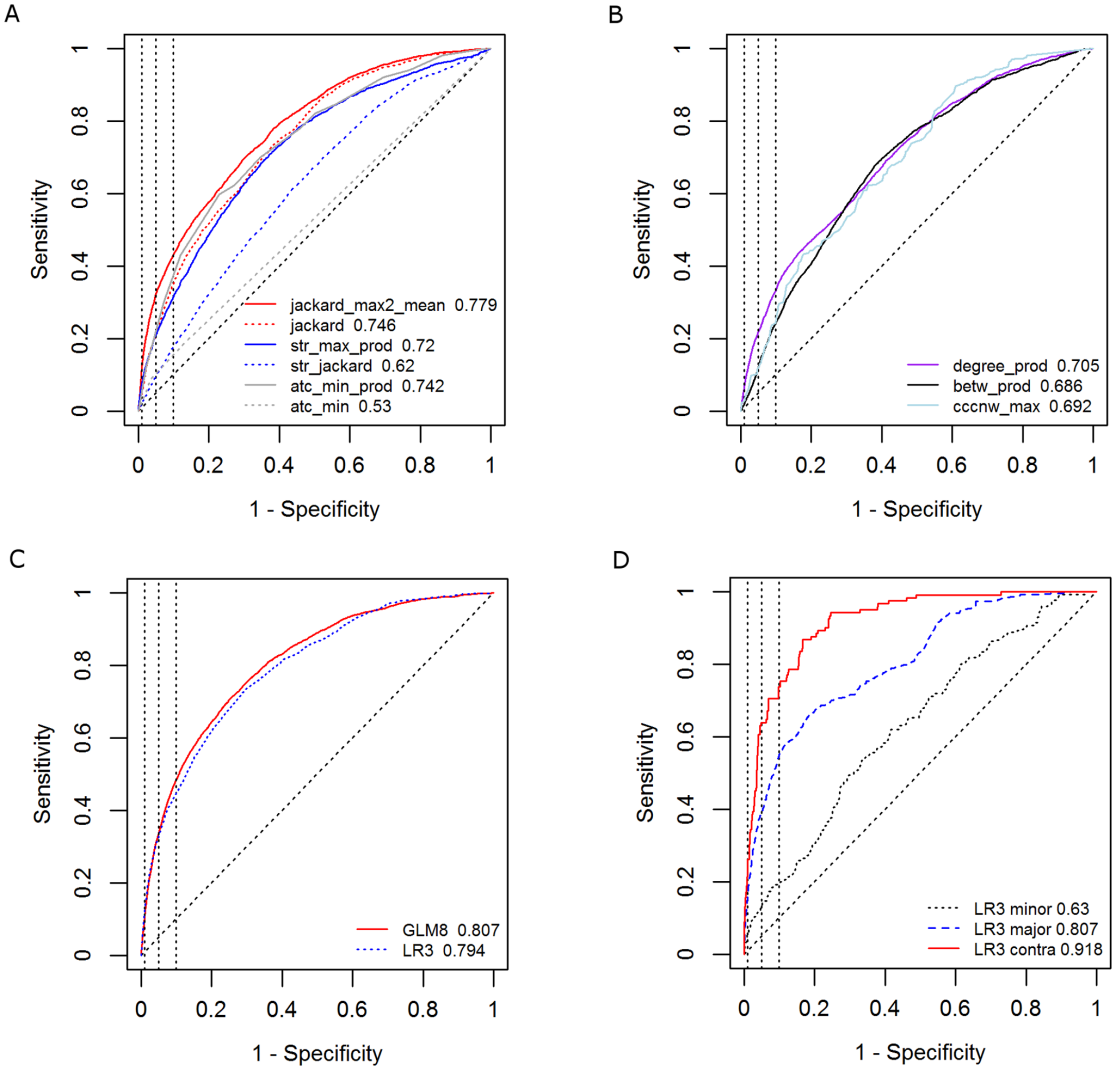
Predictive performance plots. (A) ROC curves for similarity-based covariates; (B) ROC curves for non-similarity covariates; (C) ROC curves for multivariate models; (D) ROC curves for three severity-based DDI classes: minor, major and contraindicated.

We began our analysis by performing univariate LR analysis of all covariates. Table S2 shows the results of this analysis, including parameter estimates, P values, and the AUROC achieved in the training set. Fig. 3A shows the validation set ROC curves and AUROC values for six similarity-based covariates. The best univariate performance was achieved by the 1^st^ order network covariate jackard_max2_mean (validation set AUROC 0.779), followed by 0^th^ order network covariate jackard (0.746), 1^st^ order taxonomic covariate atc_min_prod (0.742), 0^th^ order intrinsic covariate str_jackard (0.62) and 0^th^ order taxonomic covariate atc_min (0.53). Likewise, Fig. 3B shows the ROC curves and the validation set AUROCs for three non-similarity covariates.

We performed a systematic search over all subsets of covariates to identify multivariate LR and GLM models with improved prediction performance. Fig. 3C shows the ROC curves and AUROC values for two such models. A parsimonious LR model consisting of the three 1^st^ order similarity covariates (model LR3, Table S3) achieved a validation set AUROC of 0.794. A GLM model that also includes 0^th^ order and non-similarity covariates (model GLM8, Table S4) achieved a small but significant improvement over LR3 in terms of AUROC (AUROC = 0.807, p-value from comparison of LR3 and GLM8 ROC curves <0.0001). For this model, the sensitivity was 0.11, 0.34, and 0.48, given a specificity of 0.99, 0.95, and 0.90, respectively. As is often the case with predicting such rare phenomena, high specificity and sensitivity can still be associated with low PPV. At a specificity of 0.99, the PPV of the GLM8 model was 13.5% and the model lift (i.e. the fold-reduction in the search space of possible DDIs) was 9.6. The LR3 model achieved slightly higher PPV and lift values at a specificity 0.99 (15% and 10.7, respectively). Fig. 3D shows the ROC curves of model LR3 for the minor-DDI, major-DDI and contraindicated-DDI sub-networks. The respective AUROCs of these three sub-networks were 0.63, 0.81, and 0.92.

**Figure 4:**
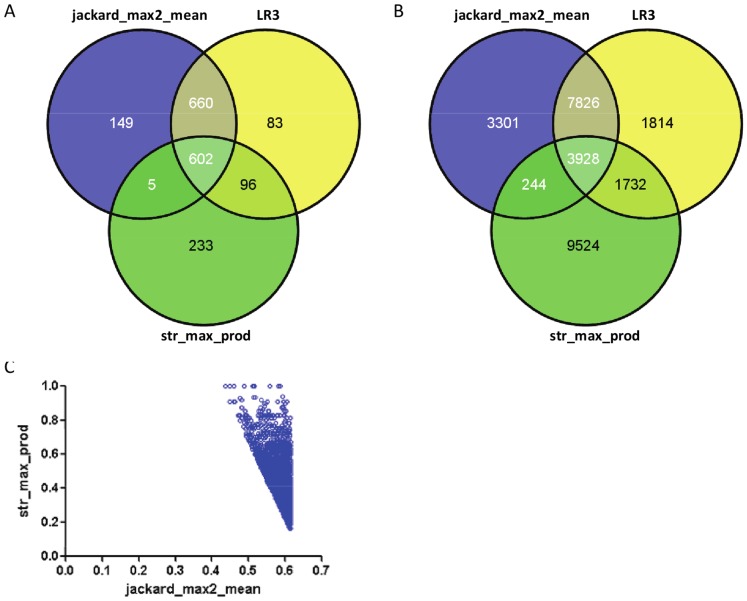
Model comparison. Analysis is based on predictions generated by the univariate models jackard_max2_mean, str_max_prod and the multivariate model LR3 when specificity of each model was fixed at 95%. (a) Three-way Venn diagram of the true positives by the three models; (B) Three-way Venn diagram of the false positives by the three models; (C) Scatter plot of jackard_max2_mean versus str_max_prod for the drug pairs predicted to be DDIs by LR3 but not by jackard_max2_mean.

The results obtained for the DDI network indicate that multivariate LR and GLM models achieved a small AUROC improvement over the best performing covariate (jackard_max2_mean) and no PPV improvement at very high specificity levels (e.g. above 99%). A close inspection of these multivariate models reveals that they are dominated by the covariate jackard_max2_mean; the remaining covariates can only slightly affect the predictions by jackard_max2_mean. As an illustration, Fig. 4, shows three-way Venn diagrams of the sets of true and false positives generated by the univariate models jackard_max2_mean, str_max_prod and the multivariate model LR3 when the specificity of each model was fixed at 0.95. As seen, nearly 88% of the true positives (Fig. 4A) and 77% of the false positives (Fig. 4B) generated by model LR3 are also generated by jackard_max2_mean. The remaining 12% of true positives and 23% of false positives generated by LR3 were drug pairs having jackard_max2_mean slightly below its 95%-specificity threshold of 0.61 and generally high values of str_max_prod (Fig. 4C).

Possibly due to this domination by one predictor, the multivariate models LR3 and GLM8 do not maximally leverage the different types of information contained by the covariates. This outcome is especially noticeable at very high specificity levels, where, as reported earlier, the multivariate PPV was no higher than the PPV produced by jackard_max2_mean. Since in real-world prospective settings a predictive model would most likely be used to generate a relatively small number of predictions that are as reliable as possible, it is natural to ask whether other methods for combining covariates could lead to improved PPV. One way to increase the PPV would be to reduce the number of false positives generated by the model. To achieve this reduction, a straightforward heuristic would be to set the training-set specificity of each covariate at a very high level and to consider only those predictions that are common to all covariates. To evaluate this heuristic we set the prediction thresholds of jackard_max2_mean and str_max_prod at their respective univariate 99^th^ percentiles in the training set of non-edges (0.756 and 0.575) and generated a set of 656 predicted drug pairs in the validation set that were above both thresholds. We found that this set of predictions contained 134 true positives, with a corresponding PPV of 20.4% - a substantial improvement over the multivariate models LR3 and GLM8.

### Prediction of DDI Type

The present study is focused on the prediction of unknown drug-drug interactions. The development of a robust method for identifying specific AEs that may be associated with certain DDIs is outside the scope of this work. For illustration purposes, we conducted an initial investigation of a basic method for suggesting potential types for the predicted DDIs. We used the “interaction description” field in the Vantage Rx database, which describes the type of interaction between two drugs. We investigated a straightforward method for predicting the DDI type associated with each predicted interacting drug pair: we examined all other known interactions involving either of these two drugs in the 2009 network (the “neighborhood types”), and identified the most common interaction types amongst them (see Methods for complete details).

**Figure 5:**
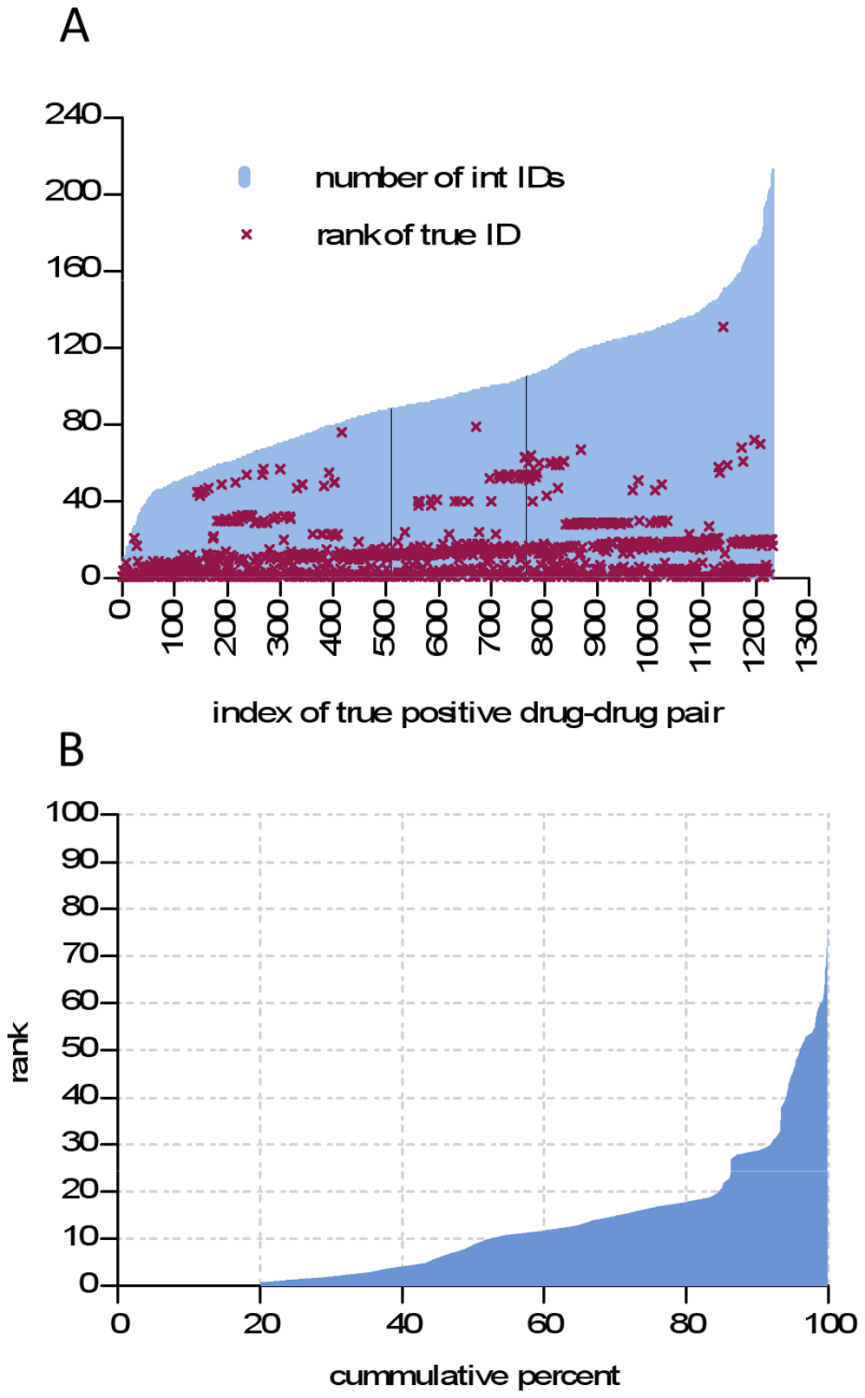
Accuracy of DDI type prediction. Analysis is based on DDI types occurring in the set of true positives predicted by the GLM model when its specificity was fixed at 0.95. (A) The true positive pairs are shown ordered according to the number of unique interaction IDs found in the pair’s neighborhood (blue bars); the rank of each pair’s true ID according to the 2012 snapshot (purple crosses) is also shown; (B) Cumulative distribution of the rank of true ID.

Fig. 5 illustrates the performance of this basic method on the set of true positives predicted by the GLM model, with its specificity fixed at 0.95. Of the 1,496 true positives predicted by the GLM model, only 1,232 had a 2012 DDI type that existed among the 2009 neighborhood types. For each of these 1,232 drug pairs, Fig. 5A shows the number of unique interaction types found in its 2009 network neighborhood and the rank of the true DDI type when the “neighborhood types” were sorted in decreasing order of frequency. The newly reported DDI types typically coincide with one of the most frequently occurring types in the network neighborhood. Fig. 5B enables a more precise description of this phenomenon by plotting the cumulative distribution of the rank of true ID. For instance, for 20% of the drug pairs, the true DDI type was the most frequent neighborhood type, for 35% of the pairs, the true DDI type was amongst the top 3, and for 43% of the pairs it was amongst the top 5. As an illustration, Table S5 shows five top-ranked triples (drug, drug, DDI type) that were correctly predicted by the above approach.

## Discussion

The proposed multivariate model for predicting unknown drug-drug interactions achieved an AUROC of 0.81 with a sensitivity of 0.48 given a specificity of 0.90. The model PPV was as high as 14%, corresponding to a ten-fold reduction in the search space of possible DDIs. A heuristic approach aimed at reducing the number of false positives resulted in a PPV of 20% and a fourteen-fold reduction in the search space. Finally, a multivariate model achieved AUROCs of 0.63, 0.81 and 0.92, respectively, for the minor-DDI, major-DDI and contraindicated-DDI sub-networks. These findings suggest that the proposed network method can be useful for predicting future reported DDIs years in advance, and that its predictive power is highest for the most severe DDIs. This proposed approach can serve as a complementary hypothesis-generation tool in supporting the work of drug safety professionals.

The proposed model included three types of covariates: network, taxonomic and intrinsic. For the taxonomic and intrinsic covariates, similarity in functional category or chemical structure was positively correlated with the similarity of DDI profiles. For network covariates, similarity in network neighborhoods was positively correlated with similarity of DDI profiles. Overall, we found that the 1^st^ order similarity-based covariates used in the model displayed a better predictive performance than the 0^th^ order covariates, implying that network structure contains useful information for the prediction of unknown DDIs. We also found that the non-similarity covariates displayed a marginal incremental contribution when combined with the similarity covariates.

Tatonetti et al. [34] have recently published a non-network based model for identifying DDIs for specific AEs. The present study differs from Tatonetti et al.’s study in several ways. First, the PPIN model is based on a drugome-level view of all DDIs, whereas each of the models proposed by Tatonetti et al. focuses on the DDIs associated with one specific AE. Second, the covariates in the PPIN model are based on the network structure formed by the known DDI relationships, whereas the covariates in Tatonetti et al.’s models are not network-based (they are computed using the AE frequencies from the Adverse Event Reporting System (AERS)). Third, to assess the model’s predictive performance, the current study uses a simulated prospective approach based on two chronologically separated snapshots of a widely used DDI database, whereas Tatonetti et al. used cross-validation within one chronological snapshot as well as comparison with computationally derived response variables.

This study has a number of limitations. The main limitation relates to the fact that no perfect “gold standard” exists in the area of drug safety. A number of studies in recent years have shown that different safety databases often disagree about the existence and the severity of drug-drug interactions [35]–[37]. The specific reference standard used in this study contains all known drug-drug interactions according to the compilers of the database, but obviously does not contain interactions that have yet-to-be discovered by the scientific community. Thus, it is not possible to tell whether the pairs not reported as interactions are confirmed true negatives or not-yet-known positives. Furthermore, the data may contain DDIs that will subsequently be removed from the database. Another limitation of the study is that it was conducted on a subset of all marketed drugs determined by data availability. Although this subset consisted of nearly nine hundred drugs, there exists the possibility of a biased sample, implying that the results would not necessarily hold for an even larger network of drug-drug interactions. Further, the validation set for this study was the set of all study-drug pairs not known to form an interaction in 2009. Some of these pairs may consist of drugs that are never or rarely prescribed together (for example, due to gender or age related reasons). Such pairs can, however, be straightforwardly filtered out by cross-referencing standard drug label information. As discussed in the Results section, the LR and GLM modeling frameworks proved satisfactory but sub-optimal for combining different sources of information. Finally, in the proposed models, causality between the model covariates and the response variable can’t be presumed due to the existence of potential confounders.

This study could be extended in several clinically important directions, such as developing a rigorous model for generating drug-drug-mechanism or drug-drug-AE predictions. Another direction is to enrich the network data with frequency and severity information on the reported DDIs to further improve predictive performance. These extensions could increase the practical value of the PPIN approach for drug-safety professionals. Due to the “gold standard” limitations mentioned earlier, a further understanding of the predictive value of the PPIN approach would be achieved by applying it to reference data from other databases.

The proposed network-based DDI prediction method can be put to immediate use, with practitioners training models using any available clinical drug interactions database, and following up on the highest scoring model predictions with thorough clinical investigations. By augmenting the existing drug safety detection tools with tools of drug safety prediction, drug interactions can be identified earlier and more accurately, reducing drug-related morbidity and mortality.

## Materials and Methods

### Network Construction

We constructed an integrated network representation of data on drugs and drug-drug interactions. In this network, nodes denote drugs and edges denote the known DDIs. The set of edges corresponds to the DDIs contained in a 2009 snapshot of the Vantage Rx database. For each drug in the network we assembled the list of chemical substructures (from DrugBank) and a list of ATC code(s). We refer to the network described above as the DDI network. We then constructed three sub-networks of the DDI network, namely those induced by the set of “minor”, “major” and “contraindicated” DDIs, respectively. We refer to these three networks as the minor-DDI, major-DDI, and contraindicated-DDI sub-networks, respectively.

### Predictive Modeling

The binary response variable *Y_ij_* denoting the presence or absence of an interaction between drug *i* and drug *j* was modeled as a Bernoulli random variable and a function of three types of covariates (Table S1): (i) Network – Covariates of the first type depend on the structure of the observed DDI network but not on the attributes of drugs; (ii) Taxonomic – Covariates of the second type depend on the structure of the observed DDI network and on the taxonomic attributes (i.e. ATC codes). As a preliminary step for creating taxonomic covariates, we computed for every pair (*drug*_1_, *drug*_2_) the minimum distance *d_ATC_*(*drug*_1_, *drug*_2_), denoting the minimum over all possible ATC positions of *drug_1_* and *drug_2_* of the length of the shortest path between *drug_1_* and *drug_2_* in the ATC taxonomy; (iii) Intrinsic – Covariates of the third type depend on the structure of the observed DDI network and on the intrinsic properties of drugs.

We began the model development by fitting all possible univariate logistic regression (LR) models, to gauge the univariate effect and significance of each covariate (Table S2). Next, we developed multivariate LR models and performed a search to optimize model fit (Akaike Information Criterion statistic) over all possible subsets of covariates. The data used to estimate each LR model consisted of the response variable *Y_ij_, i* = 1,…,855, *j* = (*i*+1),…,856 and of the corresponding values of covariates *X_ijk_* (where *k* = 1,…,8 ranges over the final set of covariates) computed from the 2009 DDI network. Thus, each pair of drugs (*i*, *j*) was represented only once in the training data for the LR model. The fitting of the LR model was carried out by maximum likelihood estimation. The statistical significance (P values) of covariates was assessed through the standard chi-square test in the LOGISTIC procedure in the Statistical Analysis System (SAS), v9.2. After the multivariate LR model was estimated, we computed the estimated probability of interaction (or, score) *pest_ij_* for each drug pair (*i*, *j*) in the validation set (the set of pairs that were non-edges in the 2009 DDI network). These model development and validation steps were also carried out for the minor-DDI, major-DDI and contraindicated-DDI sub-networks.

Finally, as a means of accounting for within-drug associations among the responses *Y_ij_*, we developed generalized linear mixed (GLM) models, which consisted of the same fixed effects as the LR models but included drug-specific random intercepts. The data used to estimate the GLM8 model consisted of the response variable *Y_ij_, i* = 1,…,856, *j* = 1,…,856 and of the corresponding values of covariates *X_ijk_* computed from the 2009 DDI network. In other words, each pair of drugs (*i*, *j*) was represented twice in the training data, once as a response for drug *i* and a second time as a response for drug *j*. This duplication was carried out to allow the correct estimation of random intercepts. Note that if the random intercepts were excluded from the model, the estimates of fixed effects and probabilities of interaction would be identical to those obtained earlier in the LR model. The fitting of the GLM models was carried out by maximum likelihood using the adaptive quadrature method in the GLIMMIX procedure in SAS v9.2 (Table S5). The “best linear unbiased predictor” (BLUP) was used to estimate random intercepts. After the multivariate GLM model was estimated, two estimated probabilities *pest_ij_* and *pest_ji_* were generated for each drug pair (*i*, *j*) in the validation set: one based on the fixed-effect estimates and the BLUP estimator for drug *i* and another based on the fixed-effect estimates and the BLUP estimator for drug *j*. Finally, we computed the predicted score for each pair (*i*, *j*) by taking the arithmetic mean of the estimated probabilities *pest_ij_* and *pest_ji_*.

We hypothesized that the validation set pairs having the highest scores would be the ones that appear as true DDIs in the 2012 snapshot of Vantage Rx. To evaluate the predictive performance we computed the validation set AUROC by comparing the scores generated for validation set pairs with the actual presence or absence of DDIs in the 2012 snapshot. In addition, we computed the model sensitivity and positive predictive value for various benchmark levels of specificity, including 0.99, 0.95 and 0.90.

### Prediction of DDI Type

To suggest likely DDI types for the predicted interactions, we employed the following straightforward method. For each predicted DDI pair (*i*, *j*) we first identified all unique DDI types corresponding to the set of edges having *i* or *j* as an end-point in the 2009 DDI network, i.e. the DDI types occurring in the network neighborhood of pair (*i*, *j*). Then, for each DDI type we computed the neighborhood frequency count, i.e. the number of times that type is encountered among the edges having *i* or *j* as an end-point in the 2009 DDI network. We then sorted the neighborhood types in decreasing order of frequency and hypothesized that one of the most frequent neighborhood types would coincide with the true DDI types observed in the 2012.

To evaluate the above method, we first identified the true positives predicted by the GLM model when its specificity was fixed at 0.95. For each such true positive we extracted the set of 2009 neighborhood interaction types sorted in decreasing order of frequency count. We computed the rank of the true interaction type (extracted from 2012 snapshot) within the set of neighborhood types and the cumulative distribution of that rank.

## Supporting Information

Table S1 Definition of covariates.
(DOCX)

Table S2 Univariate LR analysis of covariates defined in Table S1.
(DOCX)

Table S3 Multivariate LR analysis of a parsimonious model with three covariates.
(DOCX)

Table S4 Multivariate GLM model analysis of an optimal model with eight covariates.
(DOCX)

Table S5 Five illustrative top-ranked triples (drug, drug, DDI type) correctly predicted by the approach described in the sub-section “Prediction of DDI type”.
(DOCX)
