# Supplementary material for: Pharmacointeraction Network Models Predict Unknown Drug-Drug Interactions
Source: PLoS One. 2013 Apr 19;8(4):e61468. doi: 10.1371/journal.pone.0061468 (PMC3631217; doi:10.1371/journal.pone.0061468)
Supplement: Table S1 — Definition of covariates. (DOCX) [file pone.0061468.s001.docx]

**Table S1.** Definition of covariates. Variables *i* and *j* denote drugs; $N(i)$ denotes the set of neighbors of node *i* in the DDI network; *S*(*i*) denotes the set of substructures of node *i*. Definition of taxonomic covariates relies on the pre-computed ATC-based distances $d_{ATC}$ discussed in the paper.

| Covariate ID | Covariate name | Covariate definition | Additional information |
| --- | --- | --- | --- |
| 1 | jackard | $X_{1}(i,j)=J(i,j)$ | $J(i,j)=\left\vert N(i)\cap N(j)\right\vert/\left\vert N(i)\cup N(j)\right\vert$ denotes the Jackard coefficient between the neighborhood sets $N(i)$ and $N(j)$ |
| 2 | jackard_max2_mean | $X_{2}(i,j)=\text{mean} \left[\max_{k\in N(j)-\{i\}} \{J(i,k)\},\max_{k\in N(i)-\{j\}} \{J(j,k)\}\right]$ |  |
| 3 | jackard_max2_prod | $X_{3}(i,j)=\max_{k\in N(j)-\{i\}} \{J(i,k)\}\times\max_{k\in N(i)-\{j\}} \{J(j,k)\}$ |  |
| 4 | degree_prod | $X_{4}(i,j)=degree(i)\times degree(j)$ |  |
| 5 | cccnw_max | $X_{5}(i,j)=\max_{k\in N(i)\cap N\{j\}} \left\{\frac{C(k)}{\mathrm{d}\text{egree}(k)}\right\}$ | $C(k)$denotes the clustering coefficient of node $k$; see e.g. [33] |
| 6 | betw_prod | $X_{6}(i,j)=\text{betweeness}(i)\times\text{betweeness}(j)$ | betweeness of node $i$ is the number of shortest paths passing through $i$; see [33] |
| 7 | atc_min | $X_{7}(i,j)=d_{ATC}(i,j)$ |  |
| 8 | atc_min2_prod | $X_{8}(i,j)=\min_{k\in N(j)-\{i\}} \{d_{ATC}(i,k)\}\times\min_{k\in N(i)-\{j\}} \{d_{ATC}(j,k)\}$ |  |
| 9 | atc_mean2_prod | $X_{9}(i,j)=\mathop{\text{mean}}\limits_{k\in N(j)-\{i\}} \{d_{ATC}(i,k)\}\times\mathop{\text{mean}}\limits_{k\in N(i)-\{j\}} \{d_{ATC}(j,k)\}$ |  |
| 10 | str_jackard | $X_{10}(i,j)=S(i,j)$ | $S(i,j)=\left\vert S(i)\cap S(j)\right\vert/\left\vert S(i)\cup S(j)\right\vert$  denotes the Jackard coefficient between the substructure sets *S*(*i*) and *S*(*j*) |
| 11 | str_max2_prod | $X_{11}(i,j)=\max_{k\in N(j)-\{i\}} \{S(i,k)\}\times\max_{k\in N(i)-\{j\}} \{S(j,k)\}$ |  |
| 12 | str_mean2_prod | $X_{12}(i,j)=\mathop{\mathrm{m}ean}\limits_{k\in N(j)-\{i\}} \{S(i,k)\}\times\max_{k\in N(i)-\{j\}} \{S(j,k)\}$ |  |
