# Supplementary material for: Pharmacointeraction Network Models Predict Unknown Drug-Drug Interactions
Source: PLoS One. 2013 Apr 19;8(4):e61468. doi: 10.1371/journal.pone.0061468 (PMC3631217; doi:10.1371/journal.pone.0061468)
Supplement: Table S2 — Univariate LR analysis of covariates defined in Table S1. (DOCX) [file pone.0061468.s002.docx]

**Table S2.** Univariate LR analysis of covariates defined in table S1. The parameter estimates and training-set AUROC statistics were obtained by fitting univariate LR models to the 2009 data. The chi-square test based P values of all estimates were less than 0.0001.

| Covariate Name | Estimate | Training AUROC |
| --- | --- | --- |
| jackard | 10.33 | 0.89 |
| jackard_max2_prod | 11.84 | 0.98 |
| jackard_max2_mean | 15.33 | 0.98 |
| degree_prod | 6.3E-5 | 0.86 |
| cccnw_max | 1.06 | 0.84 |
| betw_prod | 9.1E-7 | 0.8 |
| atc_min | -0.22 | 0.57 |
| atc_min2_prod | -0.24 | 0.95 |
| atc_mean2_prod | -0.14 | 0.84 |
| str_jackard | 2.38 | 0.59 |
| str_jackard_max2_prod | 11.06 | 0.92 |
| str_jackard_mean2_prod | 42.98 | 0.7 |
