# Supplementary material for: Pharmacointeraction Network Models Predict Unknown Drug-Drug Interactions
Source: PLoS One. 2013 Apr 19;8(4):e61468. doi: 10.1371/journal.pone.0061468 (PMC3631217; doi:10.1371/journal.pone.0061468)
Supplement: Table S3 — Multivariate LR analysis of a parsimonious model with three covariates. (DOCX) [file pone.0061468.s003.docx]

**Table S3.** Multivariate LR analysis of a parsimonious model with three covariates. Parameter estimates and associated P values based on the chi-square test are provided. This LR model achieved a training set AUROC of 0.98.

| Parameter Name | Estimate | P value |
| --- | --- | --- |
| intercept | -8.42 | <.0001 |
| jackard_max2_mean | 11.7 | <.0001 |
| atc_min2_prod | -0.06 | <.0001 |
| str_max2_prod | 2.55 | <.0001 |
