# Supplementary material for: Pharmacointeraction Network Models Predict Unknown Drug-Drug Interactions
Source: PLoS One. 2013 Apr 19;8(4):e61468. doi: 10.1371/journal.pone.0061468 (PMC3631217; doi:10.1371/journal.pone.0061468)
Supplement: Table S4 — Multivariate GLM model analysis of an optimal model with eight covariates. (DOCX) [file pone.0061468.s004.docx]

**Table S4.** Multivariate GLM model analysis of an optimal model with eight covariates. Estimates of fixed effects and associated P values based on the t test are provided along with the estimate of covariance for the intercept and its standard error.

| Parameter Name | Estimate | P value |
| --- | --- | --- |
| intercept | -3.65 | <.0001 |
| jackard | 1.34 | <.0001 |
| jackard_max2_mean | 16.57 | <.0001 |
| degree_prod | -2E-5 | <.0001 |
| cccnw_max | -0.29 | <.0001 |
| betw_prod | 3.3E-7 | <.0001 |
| atc_min2_prod | -0.03 | <.0001 |
| atc_mean2_prod | -0.08 | <.0001 |
| str_max2_prod | 2.92 | <.0001 |
| covariance (std err) | 1.5 (0.08) |  |
