# Supplementary material for: Pharmacointeraction Network Models Predict Unknown Drug-Drug Interactions
Source: PLoS One. 2013 Apr 19;8(4):e61468. doi: 10.1371/journal.pone.0061468 (PMC3631217; doi:10.1371/journal.pone.0061468)
Supplement: Table S5 — Five illustrative top-ranked triples (drug, drug, DDI type) correctly predicted by the approach described in the sub-section “Prediction of DDI type”. (DOCX) [file pone.0061468.s005.docx]

# Table S5. Five illustrative top-ranked triples (drug, drug, DDI type) correctly predicted by the approach described in the sub-section “Prediction of DDI type”. The column “DDI type” contains a summary of the DDI’s textual description in VantageRx, showing the mechanism of interaction. The column “severity” shows the severity level of each predicted DDI.

| Drug1 | Drug2 | DDI type | Severity |
| --- | --- | --- | --- |
| amiodarone | **sunitinib** | both drugs prolong the QT interval; may lead to increased risk of ventricular arrhythmias | major |
| haloperidol | **efavirenz** | coadministration may decrease plasma concentrations of drugs that are substrates of the CYP450 3A4 isoenzyme | moderate |
| oxazepam | **diphenhydramine** | both drugs have CNS/respiratory-depressant effects; these can be additive | moderate |
| chlorpropamide | **rifapentine** | coadministration may decrease plasma concentrations of drugs that are substrates of the CYP450 2C8, 2C9, and/or 3A4 isoenzymes | moderate |
| methotrexate | **efavirenz** | both drugs have hepatotoxic effects; these can be additive | moderate |
